# Enhancing Substrate Preference of Iridoid Synthase via Focused Polarity-Steric Mutagenesis Scanning

**DOI:** 10.1021/cbe.4c00012

**Published:** 2024-05-18

**Authors:** Huifen Yu, Cuifang Ye, Yong Wang, Zhe Wang, Sai Fang, Huanhuan Jin, Lirong Yang, Wenlong Zheng, Jianping Wu

**Affiliations:** † Institute of Bioengineering, College of Chemical and Biological Engineering, Zhejiang University, No. 38 Zhe-da Road, Hangzhou, Zhejiang 310027, China; ‡ ZJU-Hangzhou Global Scientific and Technological Innovation Centre, No. 733 Jianshe 3rd Road, Xiaoshan District, Hangzhou, Zhejiang 311200, China

**Keywords:** Iridoid synthase, nepetalactol, directed evolution, enzyme engineering, substrate preference

## Abstract

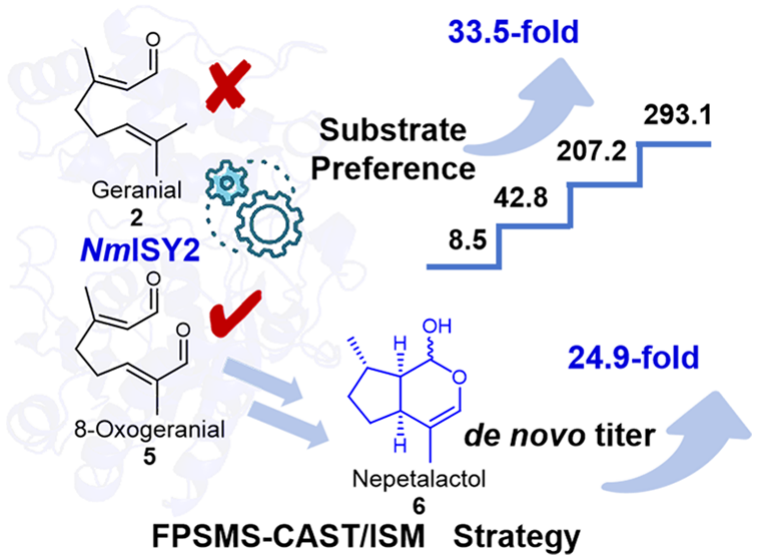

Nepetalactol serves as the scaffold for most iridoids, which exhibit a wide range of biological and pharmacological activities. Iridoid synthase (ISY) plays a crucial role in the *in vivo* synthesis of nepetalactol from 8-oxogeranial. However, the substrate promiscuity of ISY could result in a deviation of flux toward off-target routes. In this work, the substrate preference (SP, the ratio of activity for 8-oxogeranial to geranial) of ISY for nepetalactol was improved by directed evolution. First, the strategy of focused polarity-steric mutagenesis scanning (FPSMS) was performed to construct a small mutant library with *Nm*ISY2 from *Nepeta mussinii* as an object. Four amino acid residues with varying polarity and steric hindrance, including alanine, aspartic acid, serine, and arginine, were incorporated to scan hot spots. Consequently, four sites of W109, M217, K343, and W345 with a significant impact on the substrate preference of *Nm*ISY2 were found. Then, the four sites were combined by a combinatorial active-site saturation test/iterative saturation mutagenesis (CAST/ISM) strategy. As a result, the mutant W345D/K343M/W109Y (3M+) was obtained with a significantly increased SP value for **6** from 8.5 to 293.1. Molecular dynamics simulations revealed that the steric hindrance and polarity of the substrate tunnel played pivotal roles in the SP value of *Nm*ISY2. Notably, upon integration of 3M+ into *Pichia pastoris*, the *de novo* titer of **6** increased by 24.9 times, reaching 15.8 mg/L. This study offers a strategic approach to improving the substrate preference of enzymes.

## Introduction

**Figure 1:**
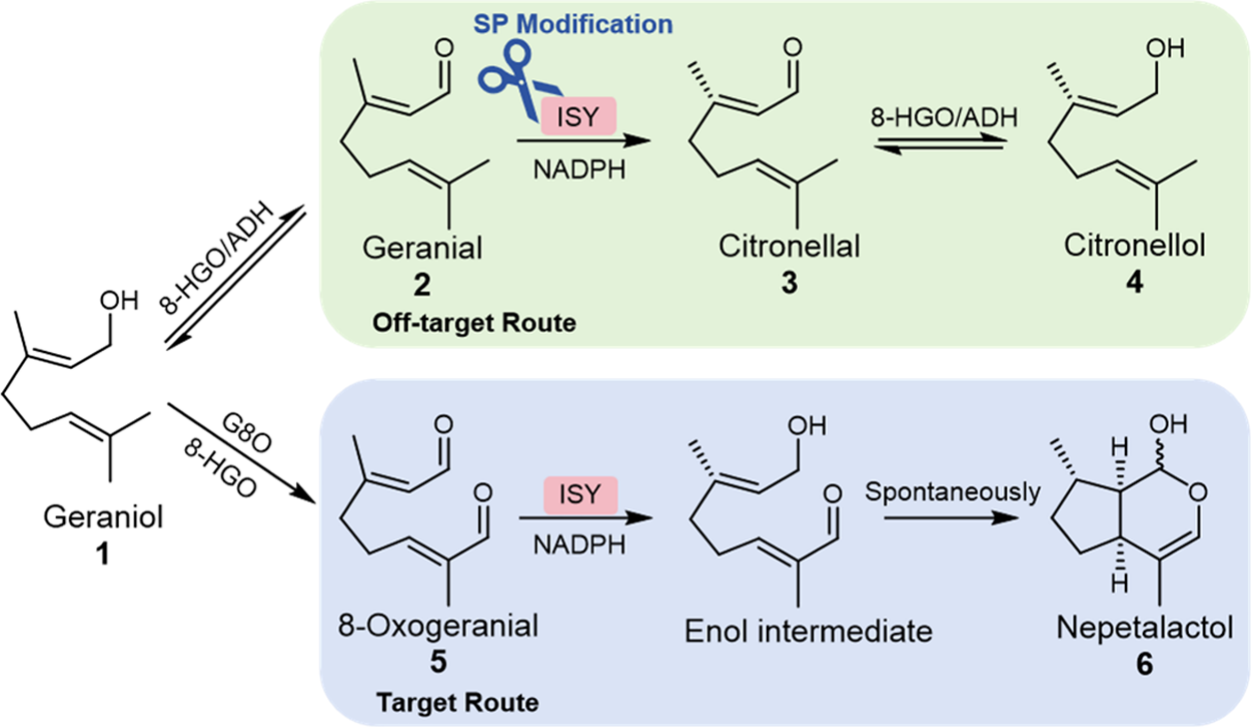
Substrate promiscuity of ISY observed in the iridoid pathway.

Nepetalactol (**6**) is a key precursor in the generation of all iridoids^1,2^ that possess a wide range of pharmacological^3^ activities. In the iridoid metabolic pathway (Figure 1), geraniol (**1**) undergoes successive catalysis by geraniol 8-oxidase (G8O) and 8-hydroxygeraniol oxidoreductase (8-HGO) to yield 8-oxogeranial (**5**).^4,5^ Subsequently, compound **5** is transformed into the unstable intermediate 8-oxocitronellyl enol under the catalysis of iridoid synthase (ISY). This unstable intermediate then spontaneously undergoes cyclization, resulting in the formation of structure **6**.^2,3,6,7^ ISY, an enzyme belonging to the superfamily of short-chain dehydrogenase/reductase (SDR),^3,8^ has been demonstrated to exhibit promiscuity with low substrate preference, resulting in the diversion of pathway flux to off-target routes.^3,9−11^ Among the off-target routes, the main byproduct is citronellol (**4**). In the metabolic pathway of **4**, ISY can reduce geranial (**2**), the oxidation products of **1**, into citronellal (**3**); then, **3** will be converted into **4** by endogenous alcohol dehydrogenase (ADH) or 8-HGO. Strategies such as regulating the expression level of enzymes could redirect part of the branch flux to the desired pathway.^12^ However, the issue of precursor diversion caused by ISY promiscuity still remains unresolved, which poses a significant challenge for both *in vivo* and *in vitro* synthesis of iridoid.^13^

Protein engineering stands as an effective approach for enhancing the substrate specificity of enzymes participating in the metabolic pathways.^9^ Leonard et al.^14^ successfully reprogrammed promiscuous enzymes acting as regulatory nodes through protein engineering, ultimately achieving overproduction and selectivity control of the diterpenoid levopimaradiene in *Escherichia coli*. Bian et al.^15^ demonstrated that terpene synthases with substrate promiscuity are widespread and enhanced the production of certain terpenoids in *Escherichia coli* exploiting engineered mutants. Lu et al.^16^ reshaped the substrate-binding pocket of phosphatase BT4131 and switched its substrate preference from glucose-6-phosphate to N-acetylglucosamine-6-phosphate, promoting the biosynthesis of N-acetylglucosamine in microbial cell factories. Sequence and structural analyses were used to investigate ISY’s substrate preference for progesterone, 2-cyclohexen-1-one, and **5**. In a study by Petersen et al.,^17^ the highly conserved phenylalanine located at the substrate-binding pocket of P5βR, an isozyme of ISY, was found to determine its substrate preference. Sandholu et al.^18^ demonstrated that Glu161, Gly162, and Asn358 of ISY from *Catharanthus roseus* (*CrI*SY) could potentially influence activity towards substrate **5**. While the results of site-directed mutagenesis indicated that the impact on substrate **5** was minimal. Notably, the examination of ISY’s substrate preference between **5** and **2** was not conducted. This inspiration led us to employ protein engineering to modify the key enzyme ISY, with the aim of enhancing its substrate preference for **5** and mitigating the off-target effects of precursors in iridoid synthesis.

Degenerate codon screening using NNK results in the construction of comprehensive enzyme libraries, which is a laborious and time-consuming process. Different strategies for constructing small and smart mutant libraries have been developed to reduce screening work, such as single-code saturation mutagenesis (SCSM), double-code saturation mutagenesis (DCSM), and triple-code saturation mutagenesis (TCSM).^19,20^ These methods are based on the analysis of the enzyme’s substrate pocket composition and the specific reactive characteristics. They involve the strategic introduction of several distinct amino acids with specific attributes at key positions, thereby substantially alleviating the screening process. It is noteworthy that these strategies typically initiate the selection of introduced amino acids based on a single attribute, such as volume or hydrophobicity, to achieve the desired catalytic function. Given that the mechanism of substrate preference remains obscure, it is unsuitable to directly employ these strategies in ISY. Hence, a scanning method considering more properties of the selected amino acid is necessary.

In this study, ISY from *Nepeta mussinii* (*Nm*ISY2) was selected as an object. And the substrate preference for **5** of *Nm*ISY2 was improved by the directed evolution strategies of focused polarity-steric mutagenesis scanning (FPSMS) and combinatorial active-site saturation test/iterative saturation mutagenesis (CAST/ISM). As a result, mutants with both increased preference and increased activity for compound **5** were obtained. Notably, the *in vivo* titer of **6** in *Pichia pastoris* increased dramatically with the integration of the mutant. This study provides a general strategy of FPSMS for modifying substrate specificity of enzymes as well as an ideal method for overcoming the bottleneck in the metabolism of natural products though enzyme engineering.

## Results

### ISY Mining, Modeling, and Docking

The widely reported ISYs from *Cantharanthus roseus* (*Cr*ISY)^3^ and *Nepeta mussinii (Nm*ISY2)^7,21^ have been proved to have the ability to convert **5** into **6**. The sequence identity between the two enzymes was only 52.64%, indicating a low degree of similarity. To identify more promising enzymes, the specific activity of *Cr*ISY and *Nm*ISY2 was determined with **5** and **2** as substrates, respectively (Table 1). As indicated in Table 1, *Cr*ISY exhibited relatively higher activity for **2** (6431.5 ± 60.7 U/g) and **5** (13363.1 ± 147.3 U/g) compared to *Nm*ISY2. However, its substrate preference was not as pronounced as that of *Nm*ISY2. We defined the ratio of activity for substrate **5** to activity for substrate **2** as the substrate preference value (SP value). A high SP value indicated that the enzyme exhibited a strong specificity for substrate **5**. The SP value of *Cr*ISY was 2.1, whereas for *Nm*ISY2, it was 8.5. As the substrate preference of ISYs was the main challenge in the iridoid metabolic pathway, *Nm*ISY2 was selected for further investigation.

**Table 1:** Specific Activity and SP Value for *Cr*ISY and *Nm*ISY2 at 30 °C^a^

	specific activity (U/g)		
enzyme	geranial (**2**)	8-oxogeranial (**5**)	SP value
*Cr*ISY	6431.5 ± 60.7	13363.1 ± 147.3	2.1
*Nm*ISY2	384.2 ± 11.6	3258.9 ± 405.9	8.5

a Reaction conditions: 200 μL of MOPS buffer (20 mM, pH 7.0) containing NADPH (200 μM), **2** (100 μM) or **5** (100 μM), and 0.5% (v/v) tetrahydrofuran (THF) as co-solvent. The concentration of the pure enzyme was adjusted according to the enzyme activity. SP value: substrate preference value, the ratio of specific activity for substrate **5** to the specific activity for substrate **2**.

**Figure 2:**
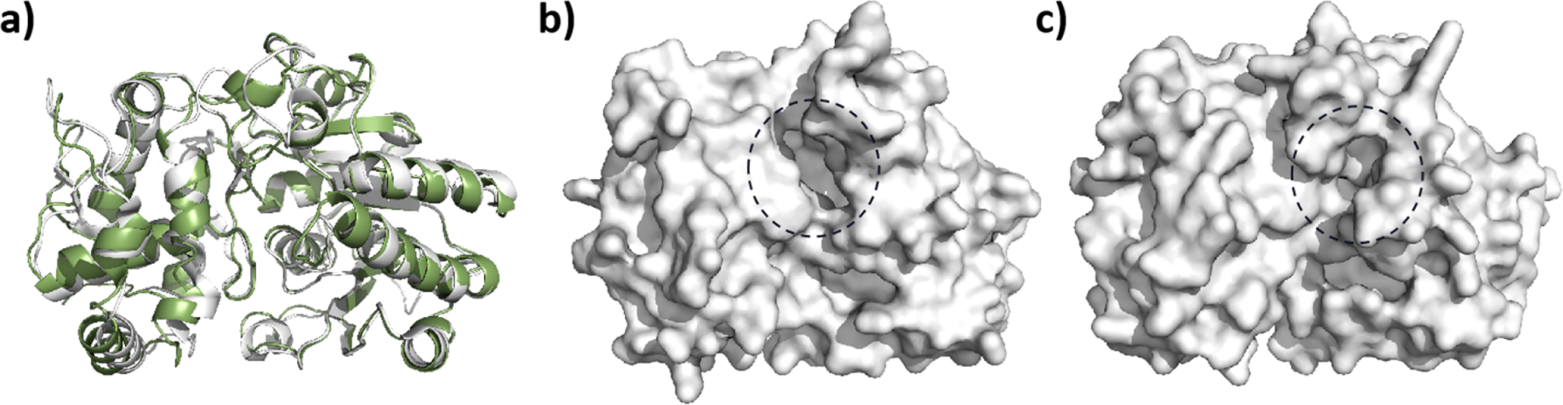
Structure of *Nm*ISY2 and *Cr*ISY^24^ (PDB ID: 5COB). (a) Superimposition of *Cr*ISY (white) and *Nm*ISY2 (smudge). (b) Surface of *Cr*ISY. (c) Surface of *Nm*ISY2.

As crystal structure was unavailable, the model of *Nm*ISY2 was built by AlphaFold 2.^22^ A total of five models were generated, and the top-ranked model was selected for subsequent analysis. Consistent with known ISYs,^23−25^ the model of *Nm*ISY2 exhibited a symmetric homodimeric structure. Each dimer comprised two domains, including the dinucleotide-binding Rossman domain and the helical capping domain, and the gap between these two domains constitutes the binding pocket. By superimposing the model of *Nm*ISY2 onto the crystal structure of *Cr*ISY^24^ (Figure 2a), it was observed that, despite their high structural similarity with an RMSD of 0.684 Å, the pocket exhibited notable differences (Figure 2b,c). *Cr*ISY was found to have a more extensive pocket compared to that of *Nm*ISY2, prompting us to hypothesize that this difference might be associated with the divergent substrate preference. Next, the model of *Nm*ISY2 was refined by MD simulation, and compounds **2** and **5** were docked to the binding pocket of *Nm*ISY2 as ligands (Figure S1), respectively.

### Construction Mutants Library by FPSMS Strategy

**Figure 3:**
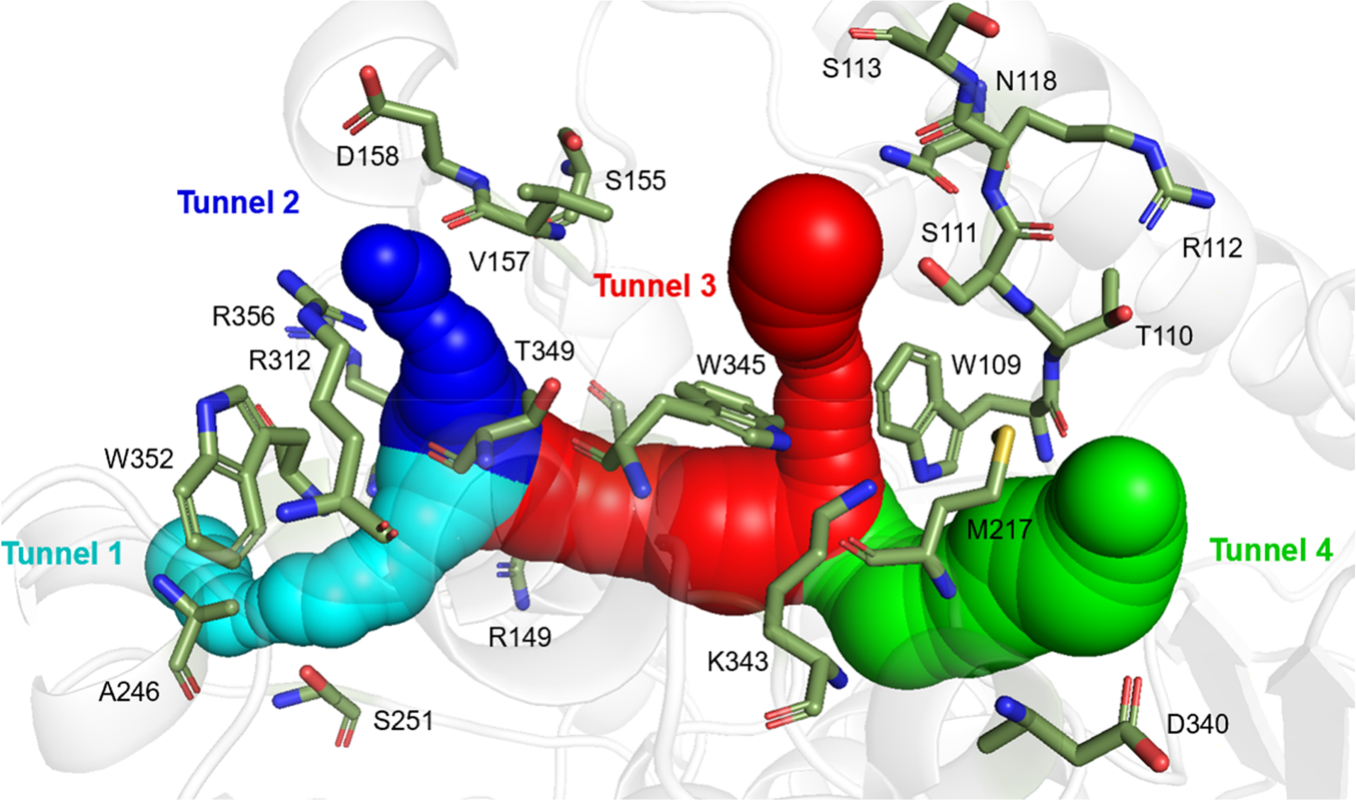
Illustration of the substrate tunnels of *Nm*ISY2. The tunnels were predicted by CAVER 3.0. Tunnel 1 is colored cyan. Tunnel 2 is colored blue. Tunnel 3 is colored red, and tunnel 4 is colored green.

As the substrate preference mechanism of ISY was not clear, the hot spots that influence the substrate preference of ISY have not been identified. Based on the structural comparison (Figure 2), we speculated that the substrate preference of ISY might be related to the binding pocket and substrate access tunnels. Substrate tunnels have been proved to be intricately linked to the substrate specificity and promiscuity of enzymes.^26^ Here, CAVER 3.0 was applied to calculate the substrate tunnels of *Nm*ISY2. As shown in Figure 3, four tunnels situated between the Rossman domain and the capping domain were predicted. They were named tunnel 1 (cyan), tunnel 2 (blue), tunnel 3 (red), and tunnel 4 (green), respectively. Furthermore, the 20 residues around the tunnels were selected to be hot spots, including W109, T110, S111, R112, S113, N118, R149, S155, V157, D158, M217, A246, S251, R312, D340, K343, W345, T349, W352, and R356 (Figure 3).

**Figure 4:**
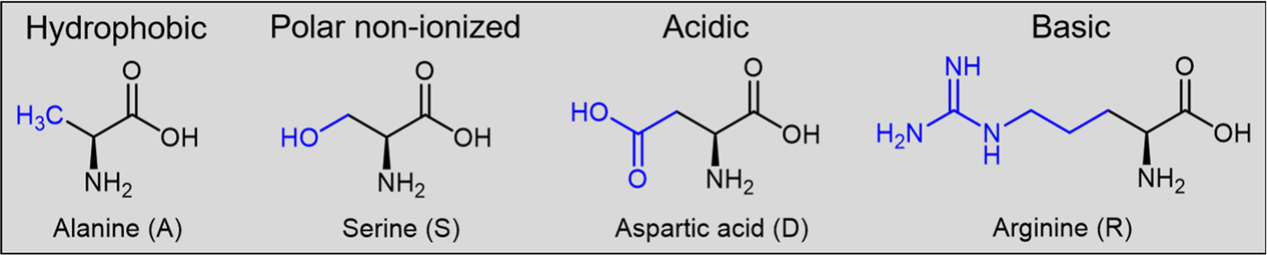
Amino acids selected for focused polarity-static mutagenesis scanning.

Saturation mutations based on the degenerate codon NNK were labor-consuming and a time cost in library construction and screening. Despite the development of strategies such as CAST/ISM, SCSM, DCSM, TCSM, and focused rational iterative site-specific mutagenesis (FRISM)^27^ to decrease screening intensity, the absence of a substrate preference mechanism in *Nm*ISY2 rendered these strategies unsuitable for application in this work. Considering the polarity and steric difference between substrates **2** and **5**, we developed a strategy named focused polarity-static mutagenesis scanning (FPSMS) to construct the mutant library. Four amino acids—alanine (A), serine (S), aspartic acid (D), and arginine (R)—with varying sizes and polarities were selected for substitution in the identified hot spots (Figure 4).

**Figure 5:**
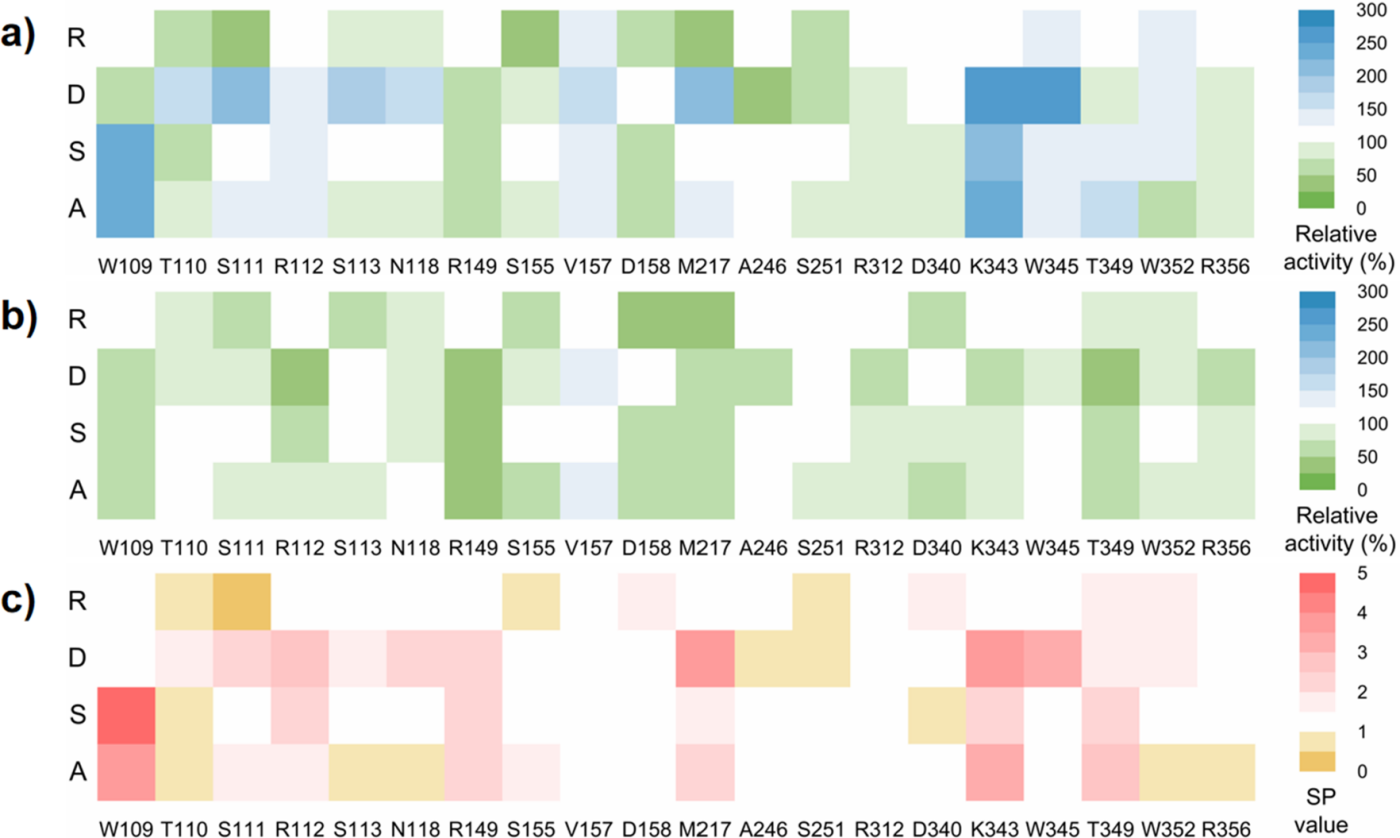
Mutability landscape of the relative activity and SP value. (a) Relative activity of mutants for **5**. (b) Relative activity of mutants for **2**. (c) SP value (ratio of activity for substrate **5** to substrate **2**) of the mutants. The relative activity of the mutants toward substrates **5** and **2** was assessed using crude enzyme.

With the implementation of FPSMS, 69 mutants were constructed. The initial screening of mutants for relative activity toward substrates **5** and **2** was conducted using the crude enzyme. In Figure 5a, mutants W109A/S, T110D, S111D, R112A/S/D, S113D, N118D, V157A/S/D/R, M217D, K343A/D/S, W345A/S/D/R, T349A/S, and W352S/D/R exhibited increased activity against substrate **5**. Conversely, for substrate **2** (Figure 5b), almost all of the mutants showed reduced activity. An interesting observation was that most sites substituted with arginine (R) did not significantly improve the activity. As arginine is an alkaline amino acid with a larger steric hindrance, we speculated that a relatively narrow tunnel and basic environment might be unfavorable for the reaction. Additionally, the substrate preference of the 69 mutants was illustrated in the SP value landscape (Figure 5c). The results revealed that mutants W109A/S, M217A/D, K343A/D, and W345D not only increased the catalytic activity for the target substrate **5** by more than twice but also decreased the activity towards **2** by at least 20%. Consequently, these seven mutants exhibited a relatively high SP value, indicating that the four sites W109, M217, K343, and W345 were key sites influencing the substrate preference of *Nm*ISY2.

Next, site-saturation mutation was performed on the four sites, resulting in mutants W109S, M217D, K343D, and W345D with significant SP value enhancements from 8.5 to 181.0, 47.1, 73.1, and 42.8, respectively (Figure S2). For the mutant W109S, specific activity showed a slight increase for substrate **5** from 3258.9 to 4523.8 U/g, while for substrate **2**, it dramatically decreased from 384.2 to 25.0 U/g, leading to a notable increase in the SP value to 181.0. For mutants M217D, K343D, and W345D, there was a 3.2-fold, 4.4-fold, and 2.4-fold increase in activity for substrate **5**, respectively. Regarding substrate **2**, the enzyme activity showed a slight decrease compared with that of WT. More interestingly, except for W109, the remaining three sites that yielded favorable outcomes were all mutated into aspartic acid, indicating that acidic environments might be more favorable for the catalysis of substrate **5**.

### Engineering *Nm*ISY2 for 8-Oxogeranial Preference by CAST/ISM

To obtain variants with a further increased substrate preference, the CAST/ISM strategy was applied to construct a combinatorial mutation library. CAST/ISM was performed with mutants W109S, M217D, K343D, and W345D as the original strains, respectively.

**Figure 6:**
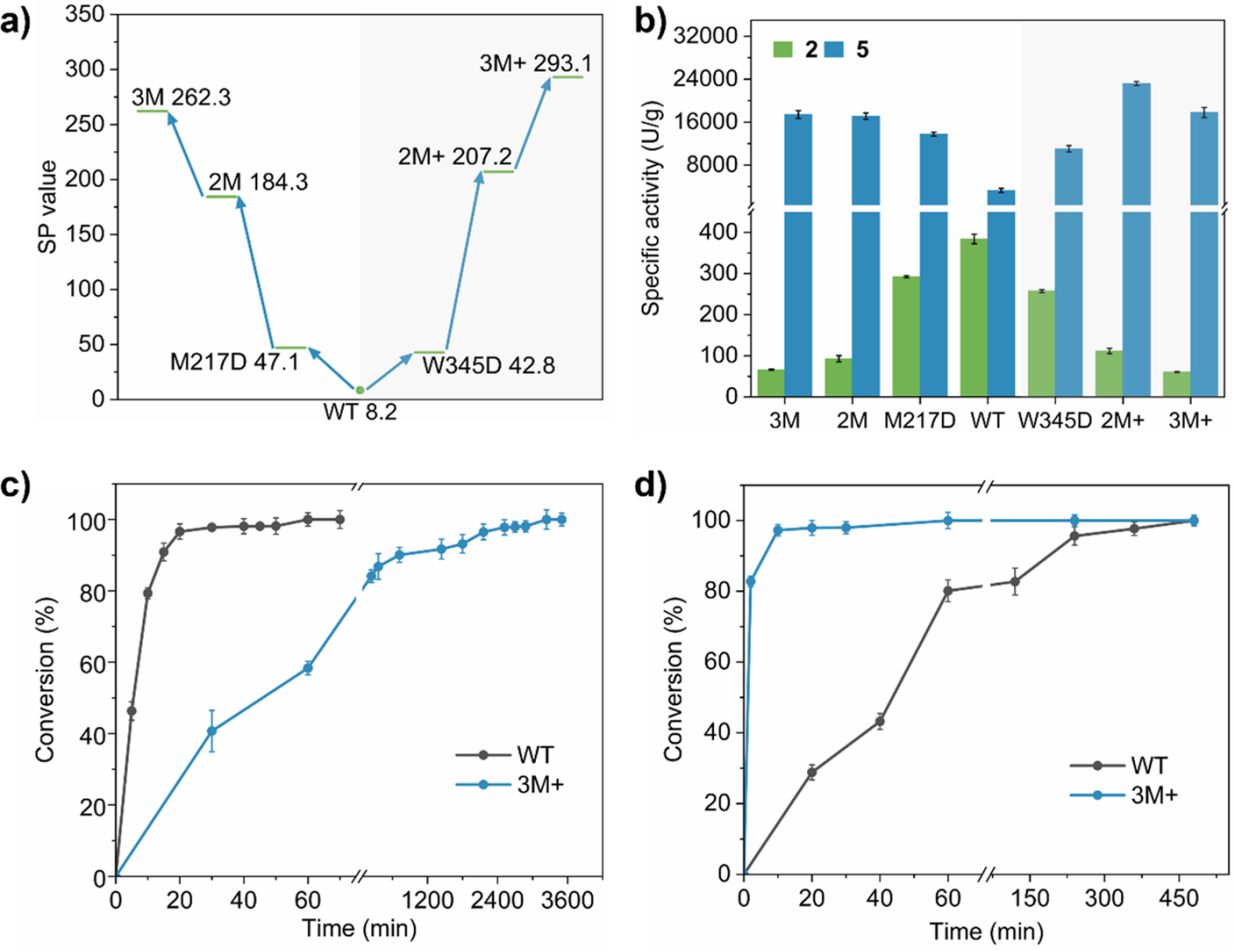
Process and results of the directed evolution of *Nm*ISY2. (a) SP value of the mutants used FPSMS strategy guided by gating elements. (b) Specific activity for substrates **2** and **5** starting from M217D or W345D. (c and d) Conversion of WT and 3M+ reacting with **2** and **5**, respectively. Reaction conditions: 30 °C, 5 mL of MOPS buffer (20 mM, pH 7.0) containing NADPH (200 μM), substrate **2** or **5** (both 100 μM), 0.5% (v/v) tetrahydrofuran (THF) as co-solvent, and purified enzyme (40 μg/mL for **2** and 2 μg/mL for **5**, respectively).

Starting from M217D (Figure 6a,b), a two-point mutation, M217D/W345D (2M), was obtained, showing a significant increase in substrate preference. The specific activity of 2M for **5** increased from 3258.9 to 17113.1 U/g, while the specific activity of **2** decreased to 92.9 U/g, resulting in an SP value of 184.3. Subsequently, 2M was used as a template for ISM. Fortunately, the SP value of three-point mutant M217D/W345D/W109Y (3M) was further improved to 262.3, with its specific activity increasing to 17410.7 U/g for **5** and decreasing to 66.4 U/g for **2**. Further ISM did not yield effective mutants with 3M as the parent.

When W345D was utilized as the starting strain for CAST/ISM (Figure 6a,b), W345D/K343M (2M+) was obtained, demonstrating an activity of 23192.0 U/g for **5** and a decrease to 111.9 U/g for **2**. The SP value of 2M+ increased to 207.2. W345D/K343M/W109Y (3M+) was finally derived when 2M+ was used as the template, showing activities of 17797.6 U/g for **5** and 60.7 U/g for **2**. The SP value of 3M+ reached 293.1, which was 33.5 times higher than that of wild-type *Nm*ISY2 (WT). Moreover, the SP value of 3M+ was 138.6 times higher than that of the widely reported *Cr*ISY.

Though the single-point mutants W109S and K343D exhibited the highest SP value and activity in the first round of site-saturation mutation, no mutants with a further improved substrate preference were obtained when using the two mutants as templates. It was noteworthy that W109 and W345 were present in mutants 3M and 3M+, respectively, highlighting the crucial role of these two sites in the substrate preference of *Nm*ISY2.

To further validate the efficacy of 3M+, we examined the reaction process of the WT and the mutant 3M+ with substrates **2** (Figure 6c) and **5** (Figure 6d), respectively, both at a concentration of 100 μM. In the reaction with substrate **2**, the WT achieved a conversion rate of >95% within 20 min, whereas the 3M+ mutant required more than 2000 min to reach a similar level of conversion. Conversely, in the reaction with substrate **5**, the WT needed over 200 min to achieve a conversion rate of >95%, while the 3M+ mutant accomplished this in just 10 min. These results indicated that the 3M+ mutant significantly enhanced its catalytic efficiency toward substrate **5**, while its ability to catalyze substrate **2** was markedly reduced compared to the WT. Furthermore, a reaction comprising both **2** and **5**, each at a concentration of 50 μM, was established to evaluate the substrate preference of 3M+ (Figure S7). Interestingly, substrate **5** almost underwent complete conversion within 10 min, whereas substrate **2** demanded over 1600 min to achieve the conversion of >95%. These results indicated that in the mixed-substrate reaction system there is a significant difference in conversion efficiency between substrates **5** and **2**, with substrate **5** having a significant advantage over substrate **2** when subjected to catalysis by the mutant 3M+.

In conclusion, through the implementation of FPSMS and CAST/ISM, mutants with improved activity and substrate preference were obtained, indicating that the FPSMS strategy was an efficient approach for constructing mutant libraries. It is worth mentioning that W109, M217, K343, and W345 were all positioned in close proximity to tunnel 3 (Figure 3). We speculated that tunnel 3 played a pivotal role as the gateway for substrate entry. And the highly conserved tryptophan pairs W109 and W345 could potentially serve as gatekeepers.

### Molecular Dynamic Simulations Reveal the Mechanism of Substrate Preference

As mentioned above, mutants 3M (M217D/W345D/W109Y) and 3M+ (W345D/K343M/W109Y) facilitated the substrate sieving and enhanced the preference for substrate **5**. The mutated sites in 3M and 3M+ were located near tunnel 3. Interestingly, these four sites were quite far from the substrate **2** or **5** (Figure S1) and might not directly participate in substrate binding. We speculated that the substrate access tunnel influenced the substrate preference of *Nm*ISY2. Therefore, 3M+ was taken as an object to reveal the mechanism underlying the improved substrate specificity by molecular dynamic simulations.

**Figure 7:**
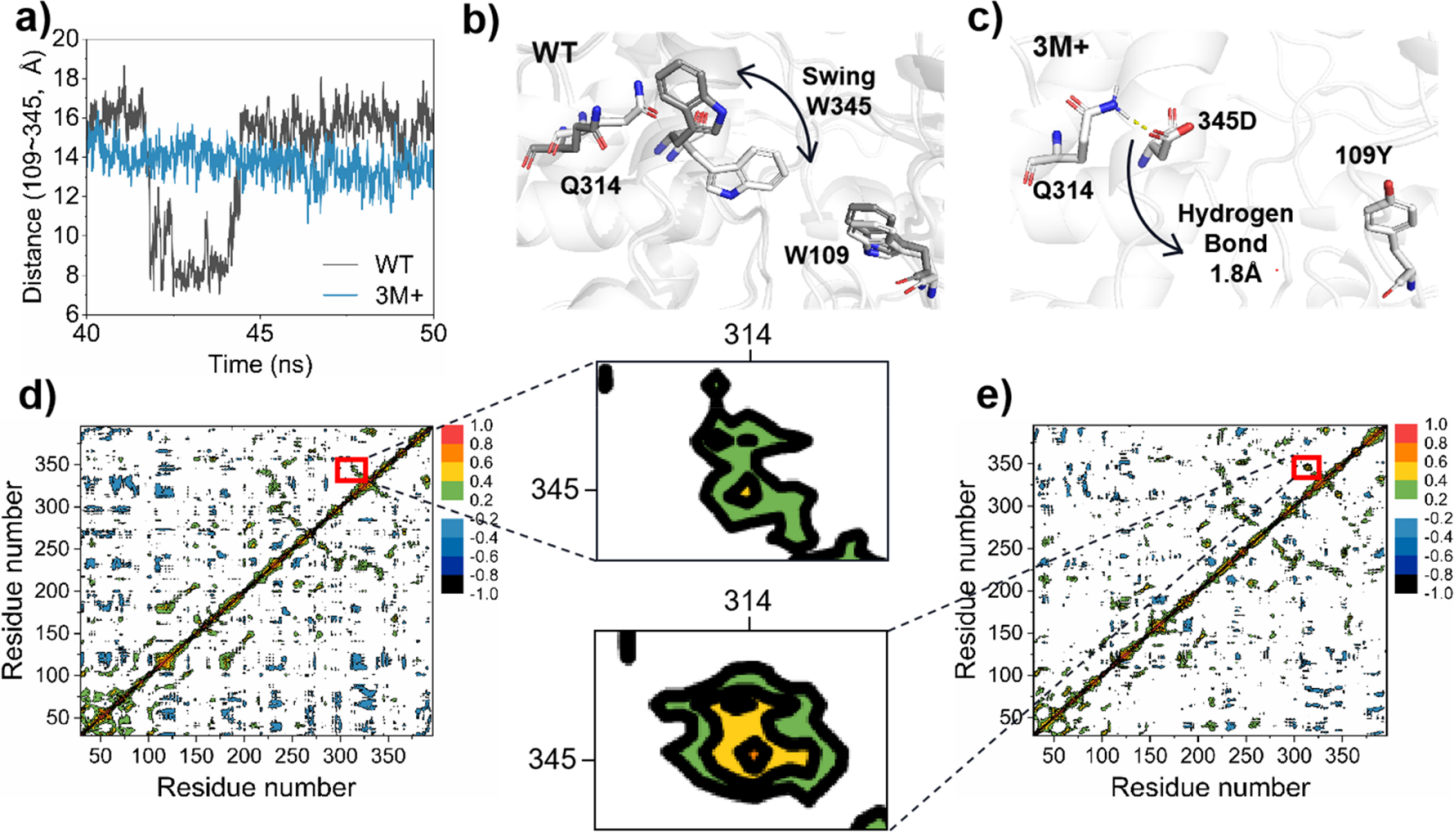
Dynamic analysis of the substrate preference mechanism. (a) Distance between residue 109 and 345. (b) The oscillation of W345 in the WT observed in the dynamic trajectory. (c) The possible hydrogen bond between Q314 and 345D in 3M+. (d) DCCM of the WT. (e) DCCM of the 3M+.

Three parallel molecular dynamics simulations were conducted for the wild type (WT) and the mutant 3M+ of *Nm*ISY2, respectively. As we suspected that W109 and W345 could potentially serve as gatekeepers of tunnel 3, the distance between W109 and W345 was investigated. An intriguing observation was made regarding the fluctuation of the distance between sites W109 and W345 in WT, while the distance between sites W109 and D345 remained stable in 3M+ (Figure 7a). Next, conformations of the molecular dynamic simulations between 40 ns and 50 ns from both WT and 3M+ were analyzed. In Figure 7b, the interaction between W345 and surrounding amino acids was weak, and W345 exhibited a swinging motion (Figure 7b). However, it was evident that in 3M+, the tryptophan gate was disrupted with the substitution of W by D at the 345 site. Furthermore, according to the statistical analysis of the distance between NE2 of residue Q314 and OD1 of residue 345D (NE2_Q314_–OD1_345D_), as well as the angle of NE2, HE22 of residue Q314, and OD1 of residue 345D (NE2_Q314_–HE22Q_314_–OD1_345D_) of the mutant 3M+, a hydrogen bond with a possibility of 90.4% might be formed between D345 and Q314 (Figure S4). This might stabilize the position of D345, resulting in a stable distance between D345 and W109 (Figure 7c). The changes were also observed in the dynamic cross-correlation matrices (DCCMs) of *Nm*ISY2 (Figure 7d,e). DCCMs depict the correlation coefficients (*C*_*ij*_) between all C_α_ atom pairs, reflecting the extent to which the fluctuation of one atom was correlated or anticorrelated with another. The *C*_*ij*_ value between residues 314 and 345 increased from 0.45 in WT to 0.68 in 3M+ due to the formation of a hydrogen bond, indicating that the positive correlation between these two residues was enhanced.

**Figure 8:**
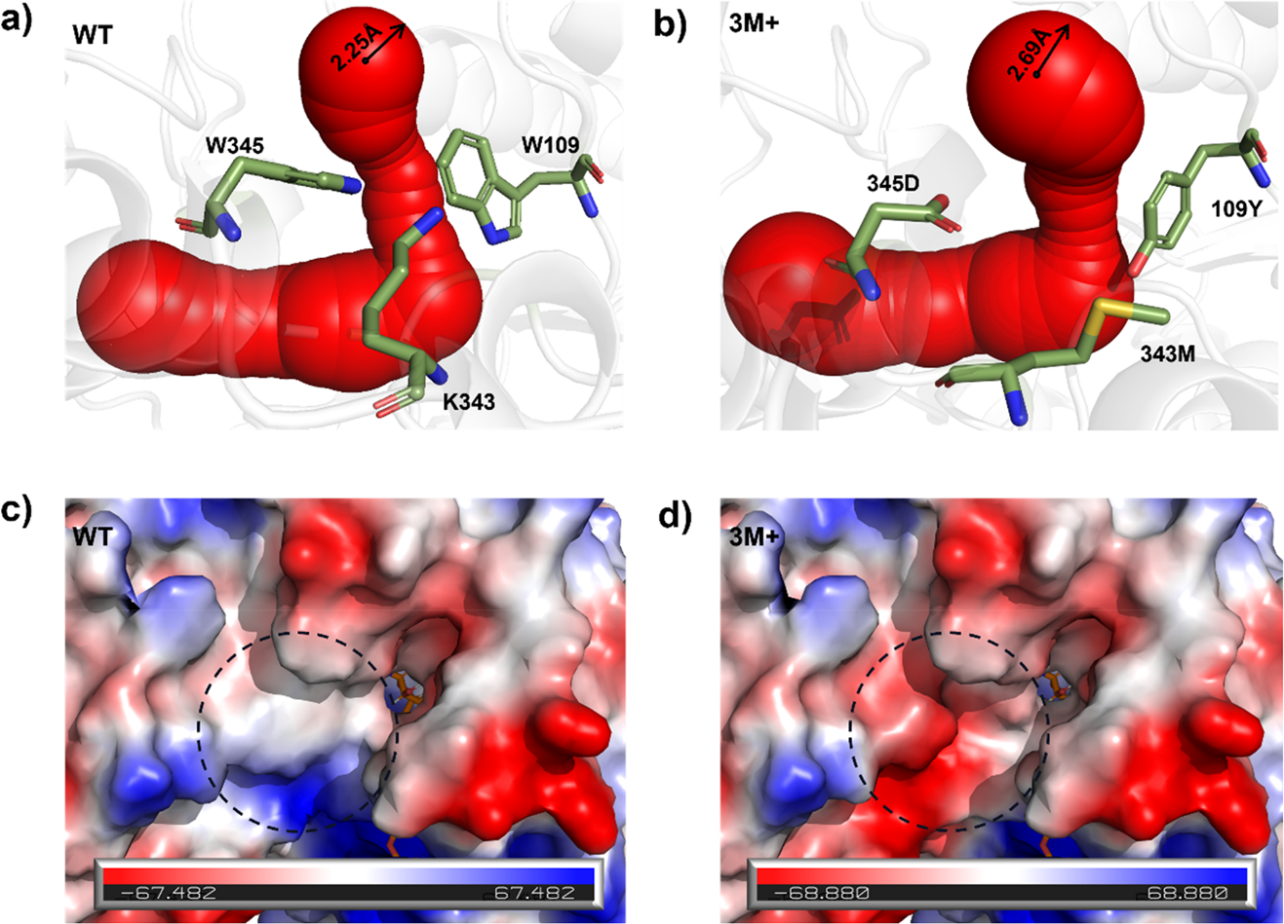
Comparison of the substrate tunnels between WT and 3M+ of *Nm*ISY2. (a) Tunnel 3 of the WT. (b) Tunnel 3 of the 3M+. (c) Electrostatic potential energy near the tunnel entrance of the WT. (d) Electrostatic potential energy near the tunnel entrance of the 3M+.

Moreover, by replacement of W with Y at the W109 site, the tryptophan gate was completely broken, resulting in a broader tunnel 3. The radius of the entrance of tunnel 3 increased from 2.25 Å in the WT to 2.69 Å in 3M+ (Figure 8a,b). Generally, the expansion of the substrate tunnel would be beneficial for the entry and exit of substrates **2** and **5**. However, the activity of the mutant 3M+ against substrate **5** increased from 3258.9 to 17797.6 U/g, but its activity against substrate **2** decreased from 384.2 to 60.7 U/g. As a crucial gatekeeper and integral component within the substrate tunnel, we hypothesized that alterations in W109 might influence the microenvironment of the tunnel, especially given the notable differences in the polarity and steric hindrance between compounds **2** and **5**. Furthermore, the electrostatic potential energy near the tunnel entrance was analyzed (Figure 8c,d). In WT, the electrostatic potential energy around the tunnel entrance ranged from zero to positive, aligning with the non-polar residues W109 and W345 and positive residue K343. This configuration facilitated the entry of compound **2** into the tunnel, given its electron-donating methyl group at C_8_. Conversely, in 3M+, the electrostatic potential energy around the entrance of the tunnel displayed a predominantly negative charge due to the substitution of Y109, M343, and D345. Given that compound **5** possessed an electron-withdrawing aldehyde group at C_8_, while compound **2** possessed a methyl group at C_8_, the negative-charge environment at tunnel 3 of mutant 3M+ theoretically increased the difficulty of compound **2** entering the active center from the tunnel, while for compound **5**, it would reduce the difficulty. Consequently, the enzyme activity and substrate preference of 3M+ for compound **5** increased simultaneously.

The analysis presented above showed that the tryptophan gating and microenvironment of the substrate tunnel played a crucial role in the substrate preference of *Nm*ISY2. To investigate the generality of the mechanism, *Nc*ISY2^21^ from *Nepeta cataria*, *Pm*MOR^23^ from *Plantago major*, and *Cr*ISY were mutated to W107Y/K343M/W345D (*Nc*ISY2-3M+), W106Y/V340M/W342D (*Pm*MOR-3M+), and W109Y/A339M/W341D (*Cr*ISY-3M+), respectively. In the case of *Nc*ISY2-3M+ and *Pm*MOR-3M+, the SP value exhibited an increase of 19.2% and 135.5%, respectively, in comparison to their respective wild types. Notably, the SP value of *Cr*ISY-3M+ displayed a decrease of 62.2%; the difference might be caused by the expansive pocket of *Cr*ISY. Above all, the results indicated that tryptophan gating and the microenvironment of the substrate tunnel were indeed key factors affecting substrate preference of ISY. Simultaneously, there were still some other factors to be explored.

### Effect of Mutant 3M+ on Synthesis of **6***in Vivo*

In order to assess the performance of the mutants *in vivo*, the WT and mutant 3M+ were integrated into *Pichia pastoris* for the synthesis of **6**. Two types of chassis (*Pichia pastoris*-1 and *Pichia pastoris*-2) served as hosts to express *Nm*ISY2. In *Pichia pastoris*-1, except for the 8-HGO located in the cytoplasm, all of the enzymes involved in the iridoid metabolic pathway were located in the peroxisome. In *Pichia pastoris*-2, all of the exogenous enzymes were situated in the peroxisome. Additionally, the OYEs of the two chassis were knocked out, as these enzymes might cause the diversion of geraniol.^28^

**Figure 9:**
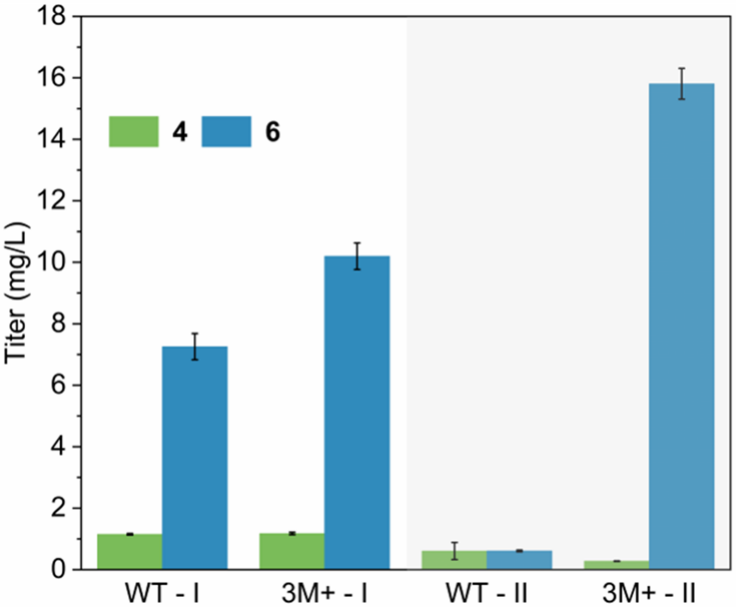
Production of **4** and **6** in recombinant *Pichia pastoris* using different subcellular localization strategies. WT-I and WT-II represented the strains integrated with the wild-type *Nm*ISY2, 3M+-I, and 3M+-II represented the strains integrated with the mutant 3M+.

With the integration of WT and 3M+ into *Pichia pastoris*-1 and *Pichia pastoris*-2, four engineering strains were constructed, including strains WT-I, 3M+-I, WT-II, and 3M+-II. In strains WT-I and 3M+-I, ISY was expressed in the cytoplasm. In the strains WT-II and 3M+-II, ISY was expressed in the peroxisome. As shown in Figure 9, the production of **6** in strains 3M+-I and 3M+-II was improved compared to the controls. However, the expression location of ISY had a significant influence on the production of **6**.

In the control WT-I, the production of **6** was 7.26 mg/L, and in the 3M+-I strains, the yield slightly increased to 10.20 mg/L. The production of the off-target product **4** did not show a noticeable change between the mutants and WT. In the control WT-II, the production of **6** was only 0.61 mg/L, approximately 10 times lower than the control WT-I. However, in 3M+-II, the production of **6** reached 15.8 mg/L that was 24.9 times that of WT-II, even 5.6 mg/L higher than 3M+-I. Additionally, the byproduct **4** of 3M+-II (0.28 mg/L) decreased by 54.1% compared to WT-II. Interestingly, the complexity of the metabolic pathway^11^ led to a decrease in the ratio of **6** to **4** production *in vivo* compared to the SP value determined *in vitro*, highlighting that the substrate preference issue of *Nm*ISY2 was more pronounced *in vivo*.

In summary, the enhanced activity and substrate specificity of promiscuous enzyme *Nm*ISY2 could boost the accumulation of **6** in *Pichia pastoris*. The observed effects were related to the subcellular localization of *Nm*ISY2 and the exogenous enzymes. Different subcellular localization strategies might impact the yield and proportion of the product. It was crucial to emphasize that, although engineering enzymes in the metabolic pathway was an efficient method to improve the production of the target product, the global regulation of the flow of matter and energy was equally important.

## Conclusion

Substrate promiscuity of ISY could result in a deviation of flux toward off-target routes. In this work, substrate specificity of the enzyme was improved for efficiency synthesis of **6**. Using the polarity-steric-based FPSMS strategy, a small mutant library of *Nm*ISY2 was obtained. W109, M217, K343, and W345, positioned close to substrate tunnel 3, were found to have a significant impact on the catalytic performance of the enzyme. CAST/ISM guided by the substrate tunnel was then applied, and a three-point mutant 3M+ (W345D/K343M/W109Y) with a 33.5-fold increase of SP value was obtained. The mechanism underlying the enhanced substrate preference was further revealed by molecular dynamics simulation and substrate tunnel analysis. The gatekeepers of W109–W345 observed by MD simulation and the microenvironment of the substrate tunnel were found to be the key factors affecting the SP value of *Nm*ISY2. The differences in substrate preferences of the triple mutants of *Nc*ISY2, *Pm*MOR, and *Cr*ISY indicated the universality of the mechanism proposed by us, but there are still more complex mechanisms waiting to be explored. Finally, the WT and the mutant 3M+ of *Nm*ISY2 were both integrated into two types of *Pichia pastoris* chassis, respectively, and the strains integrated with 3M+ promoted the accumulation of **6**. This work offered a general strategy to improving the substrate preference of enzymes and promoted the application of enzyme engineering in metabolism engineering.

## Methods

### Materials

Geranial and citronellal were obtained from AiKang (Jiangsu, China) and Topscience (Shanghai, China), respectively. 8-Oxogeranial and nepetalactol were purchased from Toronto Research Company (Toronto, Canada). NADPH was provided by Bangtai (Shenzhen, China). DNA polymerase (Primer STAR) and restriction enzymes (*Dpn* I) were purchased from TaKaRa.

### Protein Expression and Purification

The genes of *Nm*ISY2 (NCBI code: KY882236.1) and *Cr*ISY (NCBI code: AFW98981.1) with a C-terminal his-tag were codon-optimized and synthesized by GENERAL BIOL in expression of plasmid pET-28a, respectively. The genes of *Nc*ISY2 (NCBI code: A0A221J5X1.1) and *Pm*MOR (NCBI code: 5MLH_A) with an N-terminal his-tag were codon-optimized and synthesized by Yixin Biology. Then, the recombinant plasmids were transferred to *Escherichia coli* BL21(DE3) for protein expression. The strains were then kept in 25% glycerol at −80 °C.

The preserved strains were cultured overnight in LB plates with 50 μM kanamycin, and single colonies were then picked and cultured in 5 mL of liquid LB medium containing 50 μM kanamycin at 37 °C and 200 rpm for 12 h. 2 mL of the cultures was used to inoculate 1 L shake flasks containing 200 mL of liquid LB medium and kanamycin. Then, 0.5 mM IPTG would be added to induce protein expression when the OD_600_ of the cultures reached 0.6–0.8 after ∼3 h of shaking at 37v°C. Cells were harvested after 16–18 h at 18 °C and collected by centrifugation; pellets were resuspended in 50 mL of 20 mM PBS buffer (pH 7.0). Cells were disrupted on ice by a sonicator until clear and transparent. Then, all the following steps were conducted at 4 °C. The lysate was centrifuged, and the supernatant was loaded onto a 5 mL Ni-NTA column and then successively eluted with 50, 200, and 500 mM imidazole solution in 20 mM PBS buffer adjusted to pH 7.0. The eluate at 200 mM was collected and washed with a MOPS buffer (pH 7.0) using 10 kDa Amicon MWCO centrifugal filters (Millipore). The protein concentration was determined by the BSA protein assay. Fresh pure enzymes would be used for kinetic assay and specific activity determination or stored in 20% glycerol at −80 °C.

### Site-Directed Mutagenesis

Mutant construction was performed using the PCR method. The 50 μL PCR reaction system comprised 25 μL of Primer STAR, 22.5 μL of deionized water, 0.5 μL of template, and 1 μL of upstream and downstream primers. The PCR products were digested by *Dpn* I for 1 h at 37 °C and then transformed into *E. coli* BL21 (DE3) cells. Mutants conformed by PCR sequencing would be used for later screening.

### Homology Modeling, Mutant Building, Docking, and Molecular Dynamic Simulation

The model of *Nm*ISY2 was predicted by AlphaFold2 (https://colab.research.google.com/github/sokrypton/ColabFold/blob/main/AlphaFold2.ipynb). The reliability of produced models was evaluated at the site of UCLA-DOE LAB – SAVES v6.0 (https://saves.mbi.ucla.edu/). Then, the 50 ns unconstrained MD simulations were performed and repeated three times by using this model. Then, the 50 ns unconstrained MD simulations were performed and repeated three times using this model. The mutant structures were constructed with Discover Studio V4.0 using the wild-type *Nm*ISY2 as a template, the specified residues were mutated to target amino acids. The conformation with the lowest energy was selected for a subsequent analysis. The substrate and the co-enzyme NADPH were docked into the binding pocket of *Nm*ISY2 using Discover Studio V4.0. The docking procedure included the following steps: (1) The pose of substrate **2** and NADPH in PDB MLR, as well as substrate **5** and NADPH in PDB 5MLH, were superimposed into the model of *Nm*ISY2 to define the docking sites. (2) The structure was pretreated using the “Prepare Protein” module, and the ligands were energy minimized by the “Prepare Ligand” module of Discovery Studio. (3) The prepared NADPH and substrate **2** or **5** were docked into the prepared protein structures sequentially using the “CDOCKER” module. A reasonable docked pose with the lowest energy and right conformation was selected to perform MD simulations for improvement.

MD simulation was carried out using Gromacs with the force field Amber 99. The ligands **2**, **5**, and NADPH were added force field to generate a topology file by ACPYPE.^29^ The MD procedure included the following steps: (1) protein and ligand topology data and a periodic boundary were established, then water and ions were added to solvate the protein and neutralize system charges, respectively. (2) 1000 cycles of steepest descent followed by 4000 cycles of conjugate gradient minimization were used to minimize energy of the system. (3) The system was heated up to 300 K with a time step of 2 fs, then NVT and NPT were performed 50 ps in sequence with a time step of 2 fs. (4) 50 ns unconstrained simulation was carried out with a time step of 2 fs, and the output structure conformations were saved with an interval of 2 ps. The system was equilibrated if the global RMSD values were convergent. Down-sampled and RMSD-based cluster analyses were then conducted. The structure with a converged RMSD value would be used for the following mutant building and molecular docking. All of the simulations in the experiment were repeated three times.

### Tunnel Computation

CAVER 3.0 was applied to calculate the substrate tunnels of *Nm*ISY2. The default parameters were used in tunnel computing: a probe radius of 0.9 Å, a shell radius of 3 Å, and a shell depth of 4 Å.

### Reaction Process Analysis

In the single substrate reaction process assay, 5 mL systems were established in MOPS buffer (20 mM, pH 7.0) at 30 °C, including 200 μM NADPH, 100 μM substrate **2** or **5**, the co-solvent tetrahydrofuran (THF, 0.5% v/v), and purified enzyme (40 μg/mL for the reaction of **2** and 2 μg/mL for the reaction of **5**, respectively). In the mixed-substrate reaction process assay, both 50 μM substrate **2** and 50 μM **5** were added. The purified 3M+ was used at a concentration of 2 μg/mL, and all the other conditions were the same as the single substrate reaction assay. Then, 50 μL of the reaction mixture was added in 200 μL of ethyl acetate for extraction, and the supernatant was taken for GC-MS identification.

### Recombinant *Pichia pastoris* Strains and *in Vivo* Analysis

The recombinant *Pichia pastoris* strains were constructed by Professor Lian’s group. The strains were lined on YPD plates and incubated at 30 °C for 48 h. A single colony was selected and incubated in 5 mL of liquid YPD medium at 30 °C and 220 rpm for 18–20 h until the OD_600_ reached 1–2. Then, 30 μL of the cultures were transferred into a 24-well plate containing 3 mL of liquid YPD medium and incubated at 30 °C and 220 rpm for 48 h. Then, 150 μL of glucose with a concentration of 400 g/L was added when incubated for 24 h.

Then, 500 μL of the evenly mixed fermentation was transferred into 500 μL of ethyl acetate. The mixture was shaken for 1 h for extraction. After centrifugation, the supernatant was taken for GC-MS identification.

### GC-MS Analysis

The samples were analyzed by Agilent GC-MS equipped with an 8890-5977B mass selective detector (MSD). The 3 μL of the extracts was injected in pulsed splitless mode at 8.8 PSI for 2 min with an injection temperature of 250 °C. Helium was used as a carrier gas at a constant flow of 1 mL/min. A 22 min temperature gradient ranging from 70 to 180 °C was used to separate analytes over an HP-5 ms column (30 m × 0.25 mm × 0.25 μm film thickness). Citronellal was measured by 121, 139, and 154 *m*/*z*. Geranial was evaluated by following ions 69, 84, 137, and 152 *m*/*z*. Citronellol was measured by 81, 138, 123, and 156 *m*/*z*. Geraniol was estimated by monitoring 93, 123, 136, and 154 *m*/*z*. 8-Oxogeranial was detected by 84, 109, 148, and 166. Nepetalactol was measured by 135, 150, and 168 *m*/*z*.

### Spectrophotometry-Based Assays

The crude enzyme activity was determined by measuring the absorbance at 340 nm of 100-μL assays using a 96-well plate reader with time scanning mode. The temperature of the reader was set to 30 °C. The 10 μL of substrate including 0.5% (v/v) THF and 10 μL of NADPH at a final concentration of 100 and 200 μM, respectively, was added into the 96-well plate in advance, and 80 μL of the lysates (MOPS buffer, pH 7.0) appropriately diluted were then added and mixed with a microplate reader. The lysates comprising the enzyme and the substrates were incubated at 30 °C for 10 min, respectively. For specific activity determination, a 200 μL reaction system (MOPS buffer, pH 7.0) was established with 200 μM NADPH and 100 μM substrate including 0.5% (v/v) THF, and the concentration of the pure enzyme was adjusted according to the enzyme activity. For kinetic determination, a 100 μL system was established containing 400 μM NADPH, and the concentration of substrates and the pure enzyme were adjusted according to the enzyme activity. Data were collected for 1–10 min, and the NADPH consumption rates were calculated by the initial delta absorption/delta time values considering background NADPH decay and an extinction-coefficient-like value (dependent on assay volume) calculated separately.

## Supporting Information Available

Ramachandran plot; enzyme kinetic parameters; RMSD and RMSF analysis; results of GC-MS (PDF)
