# Supplementary material for: Enhancing Substrate Preference of Iridoid Synthase via Focused Polarity-Steric Mutagenesis Scanning
Source: Chem Bio Eng. 2024 May 18;1(10):826–35. doi: 10.1021/cbe.4c00012 (PMC11835258; doi:10.1021/cbe.4c00012)
Supplement: Supplementary file 1 — be4c00012_si_001.pdf [file be4c00012_si_001.pdf]

Supporting Information

Enhancing Substrate Preference of Iridoid Synthase via Focused Polarity-Steric Mutagenesis Scanning

Huifen Yu[a], Cuifang Ye[a], Yong Wang[a], Zhe Wang[a], Sai Fang[a], Huanhuan Jin[a], Lirong Yang[a,b], Wenlong Zheng[a, b*] and Jianping Wu[a, b*]

[a] Institute of Bioengineering, College of Chemical and Biological Engineering, Zhejiang University, NO.38 Zhe-da Road, Hangzhou, Zhejiang, 310027, China.

[b] ZJU-Hangzhou Global Scientific and Technological Innovation Centre, NO.733 Jianshe 3rd Road, Xiaoshan District, Hangzhou, Zhejiang, 311200, China.

[*] Email: per@zju.edu.cn, wjp@zju.edu.cn

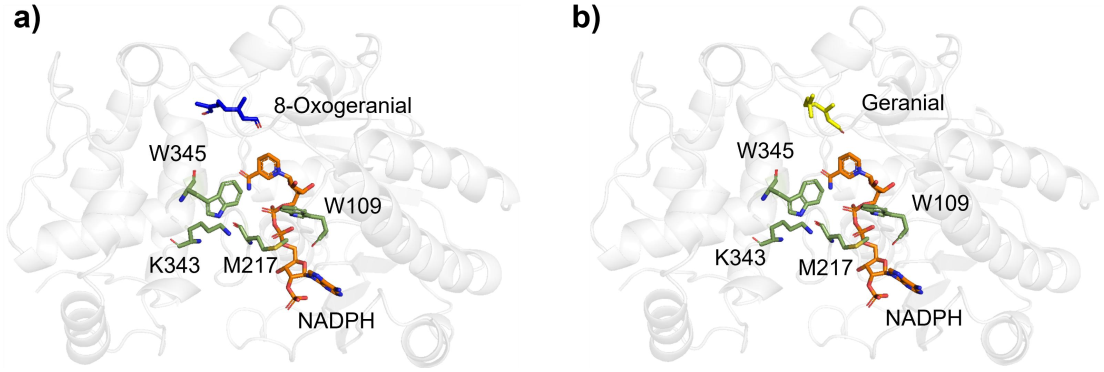

Figure S1. Relative position maps of substrates and the key residues. a) Relative position of 8-oxogeranial (**5**), NADPH, and the critical residues; b) Relative position of geranial (**2**), NADPH and the critical residues.

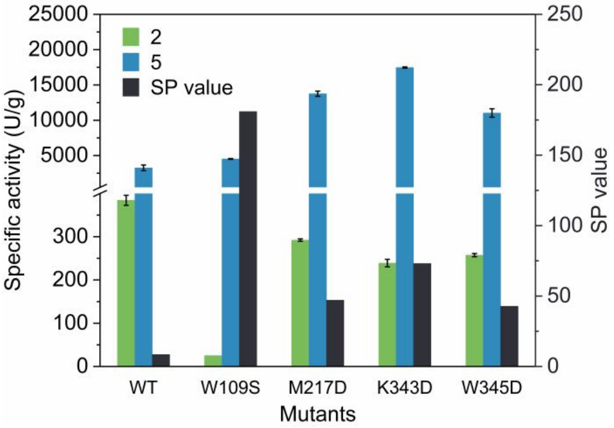

Figure S2. Single-point mutants with the most significant increase in substrate preference.

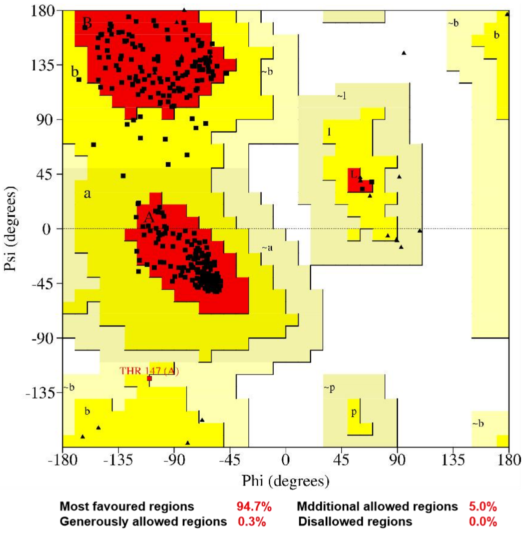

Figure S3. Ramachandran Plot of the model of *Nm*ISY2 predicted by AlphaFold2.

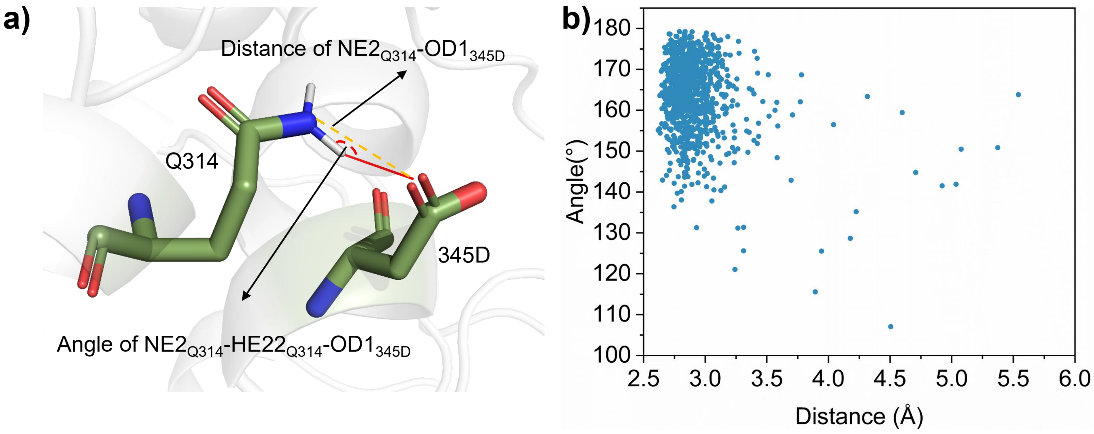

Figure S4. Hydrogen possibility maps of residue Q314 and 345D of 3M+. a) Conformation maps of residue Q314 and 345D of 3M+; b) Statistical analysis of the distance between NE2$_{Q314}$-OD1$_{345D}$ and the angle of NE2$_{Q314}$-HE22$_{Q314}$-OD1$_{345D}$.

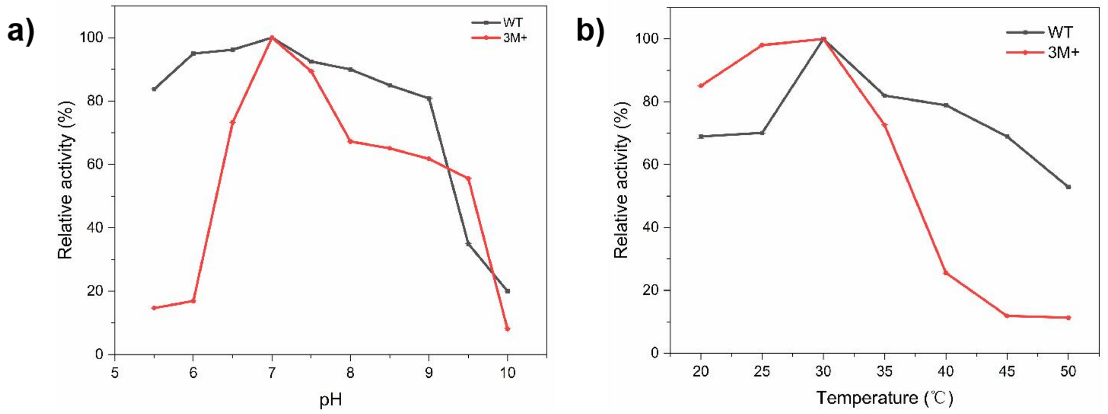

Figure S5. The optimal pH and temperature of WT and 3M+.

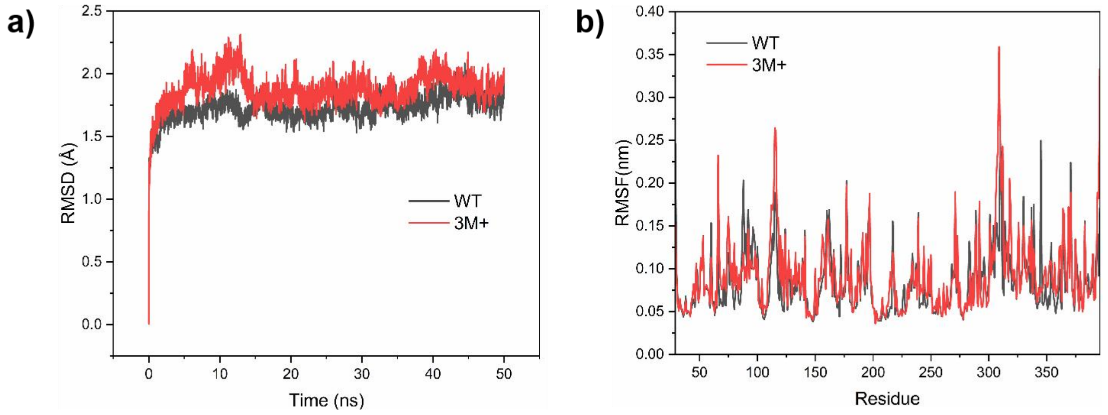

Figure S6. RMSD and RMSF of WT and 3M+.

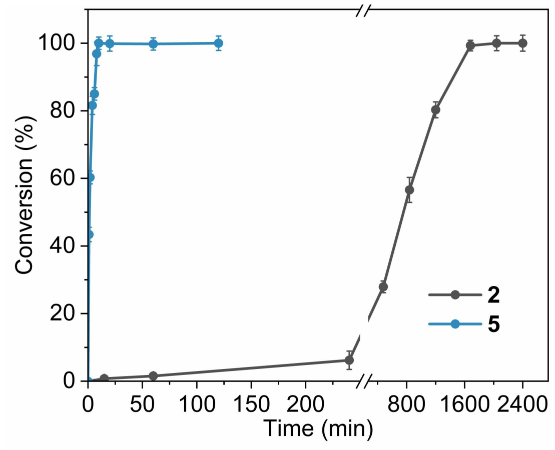

Figure S7. Process of 3M+ reacting with the mixture of **2** and **5**. Reaction conditions: 30°C, 5 mL of MOPS buffer (20 mM, pH 7.0) containing NADPH (200 μM), **2** (50 μM), **5** (50 μM), and 0.5% (v/v) tetrahydrofuran (THF) as co-solvent. The concentration of the pure enzyme was 2 μg/mL.

```
NmISY2  MSLSWWRAGAAKKRMDDDESLLVKQQQQQCVALIVGVTGLVGNSLAEMLPLPDTPGGPWKVYGVARRARP  70
CrISY   MSWWWKRSIGAGKNLPNQNKENGVCKSYKSVALVVGVTGIVGSSLAEVLKLPDTPGGPWKVYGVARRPCP  70
NcISY2  MSMNWWRDGAAKKRMD--ESSAVKLQQQQCVALIVGVTGLVGNSLAEMLPLSDTPGGPWKVYGVARRPRP  68
PmMOR   ---MSWWWAGAIGAAKKRSDEDEALPKHSSVALIVGVTGIVGNSLAEILPLADTPSGPWKVYGVARRPRP  67

NmISY2  SWNEDQPMTYISCDVSNTGEVEAKLSPLSDVTHIFYATWTSRSTEEENCEANGKMLKNVLDAMIPNCPNL  140
CrISY   VWLAKKPVEYIQCDVSDNQETISKLSPLKDITHIFYVSWIGS----EDCQTNATMFKNILNSVIPNASNL  136
NcISY2  SWNEDHPINYISCDVSNTAEVEAKLPPLSDVTHIFYATWTSRSSEEENCEANGKMLKNVLDTMIPNCPNL  138
PmMOR   AWNEDNPINYIRCDISDPKDTQEKLSPLTDITHVFYVTWANRSTEVERCEANGKMLKNVLDVVIPNCPDL  137

NmISY2  KHICLQTGRFHYVASVVDWKINGSHDTPLTEDLPRLKTNNFYYTQEDILLEEVKRKEGLTWSVHRPGTIF  210
CrISY   QHVCLQTGIKHYFGIFEEGSKVVPHDSPFTEDLPRLNVPNFYHDLEDILYEETG-KNNLTWSVHRPALVF  205
NcISY2  KHICLQTGRFHYVASVVDWKINGSHDTPLTEDLPRLNTNNFYYTQEDILLEEVKRKEGLTWSVHRPGTIF  208
PmMOR   KHISLQTGRKHYVGPFELIGKIETHDPPFTEDLPRLKFDNFYYTQEDLLFEEVEKKEGLTWSVHRPGNIF  207

NmISY2  GFSPYSMMNLVGTLCVYAAICKQEGAVLRFPGCKGAWDGHSDCADADLIAEQQIWAALDPHAKNQAFNVS  280
CrISY   GFSPCSMMNIVSTLCVYATICKHENKALVYPGSKNSWNCYADAVDADLVAEHEIWAAVDPKAKNQVLNCN  275
NcISY2  GFSPYSMMNLVGTLCVYAAICKQEGAVLRFPGCKGAWDGYSDCADADLIAEHYIWAALDPHAKNQSFNVS  278
PmMOR   GFSPYSMMNLVGTLCVYAAICKHEGKVLRFPGCKAAWDGYSDCSDADLIAEHHIWAAVDPYAKNEAFNVS  277

NmISY2  NGDLFKWKHLWKVLADQFGVECG--DYEEGQQLRLQDVMKDKGPVWDKIVAENGLSNTKLEDVGKWWFSD  348
CrISY   NGDVFKWKHIWKKLAEEFGIEMVG-YVEGKEQVSLAELMKDKDQVWDEIVKKNNLVPTKLKEIAAFWFAD  344
NcISY2  NGDVFKWKHLWKVLAEQFGVECGGYEYEEGQQVRLQDVMKDKGPVWDKIVRENGLSNTKLEDVGKWWFSD  348
PmMOR   NGDVFKWKHFWKVLAEQFGVECG--EYEEGENLKLQDLMKGKEPVWEEIVRENGLASTNLEDVAVWWFSD  345

NmISY2  TILWNECRLDSMNKSKEHGFLGFRNSKNCFLYWIHKVKAYNLVPSTYTLE  398
CrISY   IAFCSENLISSMNKSKELGFLGFRNSMKSFVSCIDKMRDYRFIP------  388
NcISY2  TILWNECRLDSMNKSKEHGFLGFRNSKNCFLYWIHKLKAYKIVPSSTIS-  397
PmMOR   AVLDIPCPLDSMNKSKEHGFLGFRNSKNSFISWIDKAKAYKIVP------  389
```

Figure S8. Sequence alignment of *Nm*ISY2 and ISY from other sources.

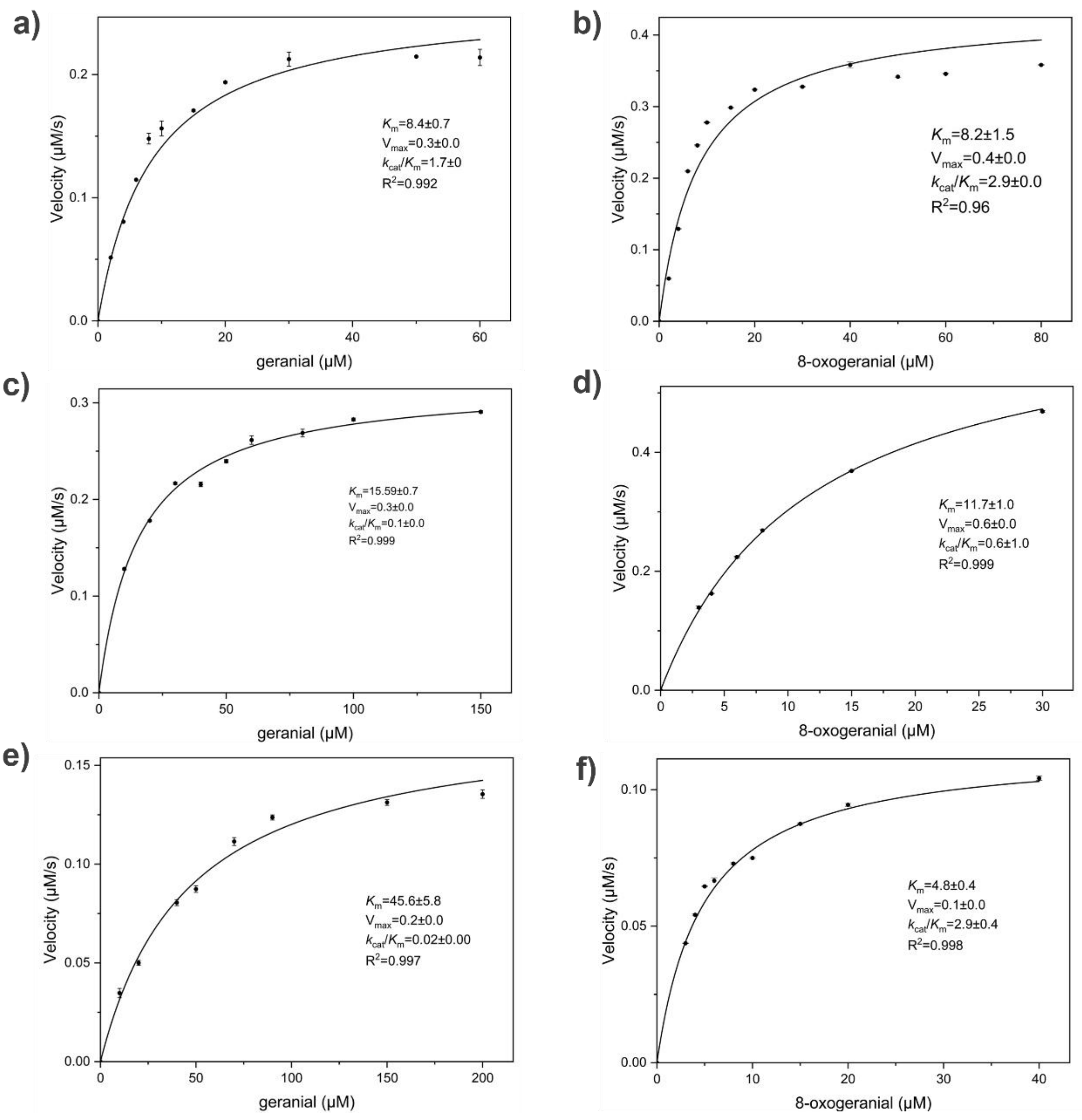

Figure S9. The Michaelis-Menten plots of geranial (**2**) and 8-oxogeranial (**5**). a) and b) were for *Cr*ISY; c) and d) were for the wild-type of *Nm*ISY2 (WT); e) and f) were for 3M+.

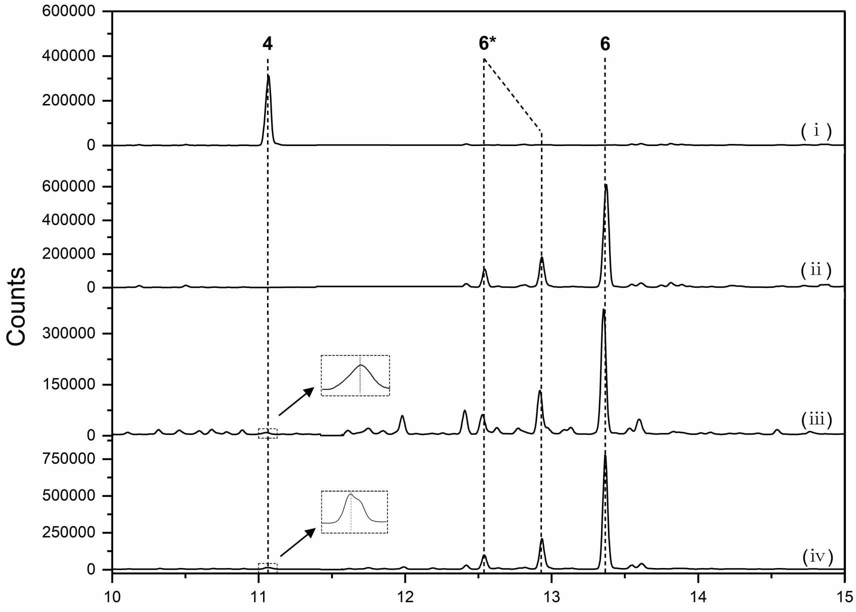

Figure S10. GC-MS analysis of the *in vivo* experiments. (i) represents the the standard **4**; (ii) represents the the standard **6**, compound **6*** (iridodials)[1,2] represents the open dialdehyde form of **6**, the sum of the peak area of **6** and **6*** was used for quantitative analysis; (iii) represents the analysis of the extracts of the 3M+-I fermentation; (iv) represents the analysis of the extracts of the 3M+-II fermentation"

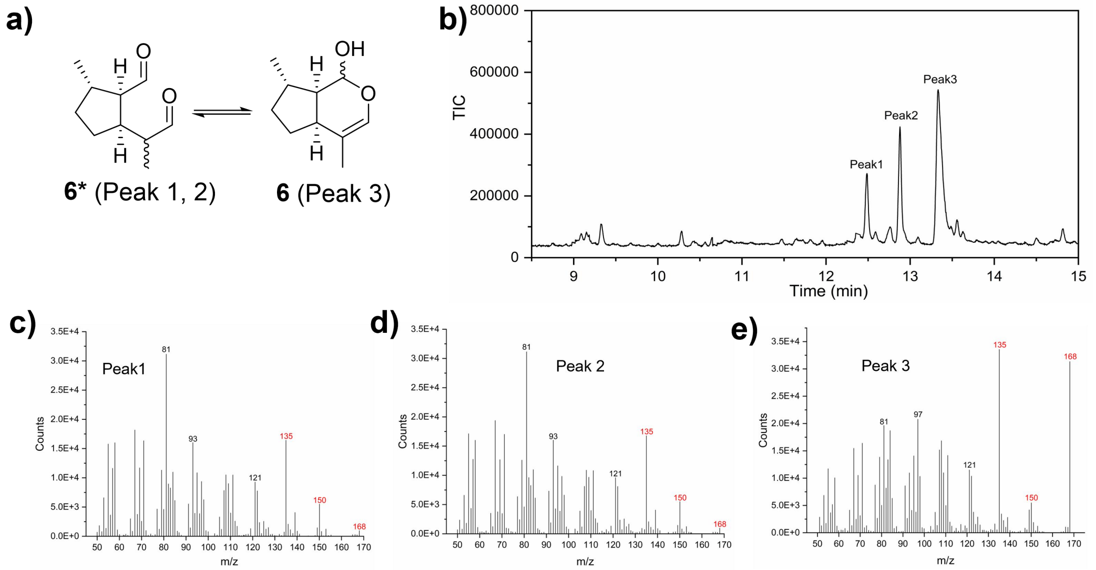

Figure S11. Full characterization of the product **6**. a) Equilibrium of **6** and its open dialdehyde form **6*** (iridodials)[2]; b) Total ion chromatogram of the product **6** by GC-MS; c), d), e) represents the mass spectra of peak1, peak2, and peak3 determined by GC-MS, respectively.

Table S1. Specific activity of the wild-type and their three-point mutants

Enzyme	Specific activity (U/g)		SP
	Geranial (**2**)	8-Oxogeranial (**5**)	
*Cr*ISY-WT	6431.5±60.7	13363.1±147.3	2.1
*Cr*ISY-3M+	3644.8±28.7	2860.3±9.6	0.8
*Nc*ISY2-WT	13.2±0.7	657.7±12.0	50.0
*Nc*ISY2-3M+	24.9±3.8	1482.8±326.2	59.6
*Pm*MOR-WT	36.4±2.9	779.7±33.5	21.4
*Pm*MOR-3M+	20.1±1.1	1012.6±111.3	50.4

References

1. Geu-Flores, F.; Sherden, N. H.; Courdavault, V.; Burlat, V.; Glenn, W. S.; Wu, C.; Nims, E.; Cui, Y.; O'Connor, S. E., An alternative route to cyclic terpenes by reductive cyclization in iridoid biosynthesis. *Nature* **2012,** *492* (7427), 138-142.
2. Alagna, F.; Geu-Flores, F.; Kries, H.; Panara, F.; Baldoni, L.; O'Connor, S. E.; Osbourn, A., Identification and Characterization of the Iridoid Synthase Involved in Oleuropein Biosynthesis in Olive (Olea europaea) Fruits. *J Biol Chem* **2016,** *291* (11), 5542-5554.
